# Distribution, Transition and Thermodynamic Stability of Protein Conformations in the Denaturant-Induced Unfolding of Proteins

**DOI:** 10.1371/journal.pone.0091129

**Published:** 2014-03-06

**Authors:** Liujiao Bian, Xu Ji

**Affiliations:** College of Life Science, Northwest University, Xi’an, Shaanxi, China; Aligarh Muslim University, India

## Abstract

**Background:**

Extensive and intensive studies on the unfolding of proteins require appropriate theoretical model and parameter to clearly illustrate the feature and characteristic of the unfolding system. Over the past several decades, four approaches have been proposed to describe the interaction between proteins and denaturants, but some ambiguity and deviations usually occur in the explanation of the experimental data.

**Methodology/Principal Findings:**

In this work, a theoretical model was presented to show the dependency of the residual activity ratio of the proteins on the molar denaturant concentration. Through the characteristic unfolding parameters *k*_i_ and Δ*m*_i_ in this model, the distribution, transition and thermodynamic stability of protein conformations during the unfolding process can be quantitatively described. This model was tested with the two-state unfolding of bovine heart cytochrome *c* and the three-state unfolding of hen egg white lysozyme induced by both guanidine hydrochloride and urea, the four-state unfolding of bovine carbonic anhydrase *b* induced by guanidine hydrochloride and the unfolding of some other proteins induced by denaturants. The results illustrated that this model could be used accurately to reveal the distribution and transition of protein conformations in the presence of different concentrations of denaturants and to evaluate the unfolding tendency and thermodynamic stability of different conformations. In most denaturant-induced unfolding of proteins, the unfolding became increasingly hard in next transition step and the proteins became more unstable as they attained next successive stable conformation.

**Conclusions/Significance:**

This work presents a useful method for people to study the unfolding of proteins and may be used to describe the unfolding and refolding of other biopolymers induced by denaturants, inducers, *etc*.

## Introduction

Studies on the unfolding and refolding of proteins have been attracting the attention of researchers in the fields of biochemistry, biophysics and structural biology for the past decade, and they play an important role in gaining an understanding of the relationship between the structure of proteins and their functions [1]–[5]. To date, the conformational transition of proteins mainly originates from studies on the unfolding and refolding of proteins induced by denaturants, pH, heat, *etc* [6], [7]. Of these factors, denaturants may be the most important. Through the conformational transition of the proteins induced by denaturants, valuable information has been obtained on the self-organization, function, misfolding and aggregation of proteins [8]–[10].

In fact, the denaturant-induced conformational transition of proteins can be considered the result of the interaction between the protein and denaturant molecules. Through studies on conformational transitions, it is now believed that globular proteins are usually unfolded through one or several denatured but compact equilibrium intermediate(s) [11], [12], and they may exist in four different types of conformation states: native states (N), molten globule states (MG), pre-molten globule states (PMG), and completely unfolded states (U) [13], [14]. One of the most characteristic features of the partially folded conformations (MG and PMG) is their combination of properties that are typical of the N and U states. Proteins in MG states have a globular structure that is typical of native globular proteins, and they may have a native-like secondary structure and a native-like folding pattern; while proteins in PMG states have no rigid tertiary structure and are characterized by a considerable secondary structure, although it is much less pronounced than that of N or MG state proteins. Proteins in this state are considerably less compact than those in the MG or N states, but they are still more compact than those in the random coil [15]–[19].

Over the past several decades, four approaches have been proposed to describe the interaction between proteins and denaturants. All of these approaches use the following general relationship to describe the unfolding free energy (Δ*G*_U_) of the protein:
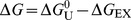
(a)where Δ*G*_U_^0^ indicates the unfolding free energy of the protein in the absence of denaturants and Δ*G*_EX_ denotes the difference in the excess free energy of the interaction between the denaturant and the native or denatured protein in its conformational transition. The first approach is the purely phenomenological linear extrapolation method shown in Eqn. (b):
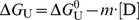
(b)where m indicates the slope of the dependence of ΔGU on the molar denaturant concentration ([D]) in denaturation solution. Therefore, the unfolding free energy (Δ*G*_U_^0^) of the protein in the absence of denaturants can be obtained by the linear extrapolation of the Δ*G*_U_ values observed in the denaturation transition zone when the denaturant concentration ([D]) is zero [20]. The second approach relies on the free energy of transferring amino acids and peptides from water into the aqueous denaturant solution [21]–[23]. These data can be used in conjunction with the amino acid composition of the protein to derive a measure of the change in the solvent-accessible surface area with differing denaturant concentrations [23]. The third approach views the denaturation of the protein as the result of the binding of the denaturant by the native and denatured protein [23]:
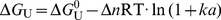
(c)where k indicates the binding constant of the protein to the denaturant, Δ*n* denotes the difference in the number of binding sites on the surface of the native and denatured protein, and *R* and *T* represent the gas constant and the absolute temperature, respectively. The fourth approach is similar to the third approach. In this method, a binding formalism is used in its mathematical development, but it treats the act of denaturation more like solvation than binding [24]–[27]. A key distinction between the solvent exchange approach and the denaturant binding approach is the formal inclusion of the solvent in the interaction between the protein and denaturant in the former. Of these four approaches, the most often used is the linear extrapolation approach.

However, it should be realized that although these approaches have been used to describe the conformational transition of proteins induced by denaturants, some ambiguity and deviations usually occur in the explanation of the experimental data. For example, for the linear extrapolation method, although this method has been widely used to derive the unfolding free energy (Δ*G*_U_^0^) of the protein in the absence of denaturants [28]–[32], the following problems usually exist. (i) The method is semi-empirical and lacks an authentic theoretical basis. The linear relationship between the denaturant concentration ([D]) and the unfolding free energy (Δ*G*_U_) of the protein is only an experimental phenomenon; it is not based on any authentic theory and is not certain to exist over the whole denaturant concentration range. Therefore, whether the linear extrapolation method is reasonable to describe the interactions between proteins and denaturants is a controversial issue. (ii) Theoretically, the conformational transition of protein should be described by the thermodynamic equilibrium constant of its conformational transitions rather than by the concentration ratio (*C*_A_/*C*_B_) of its two adjacent conformation states (A and B). For a given unfolding system, when temperature and denaturant concentration are known, the unfolding free energy (Δ*G*_U_) of the proteins only depends upon the stability of its two adjacent conformation states and has no relation to the denaturant concentration. However, the concentration ratio (*C*_A_/*C*_B_) in the presence of different denaturant concentrations is used in this method and it is involved not only in the temperature and the characteristics of the denaturant but also in the denaturant concentration. (iii) Some inconsistent and even paradoxical conclusions frequently occur using this semi-empirical method. For example, in most cases, the unfolding free energy (Δ*G*_U_^0^) of the protein in the absence of denaturant, which is derived from one kind of denaturant, is very different from another type of denaturant. Similarly, some ambiguity and deviations also exist in the other three methods. For example, the values of parameter *m* derived from the denaturant binding method are always approximately 10–100% less than those derived from calorimetric studies of urea and guanidine hydrochloride solutions [33]. Therefore, it is necessary and urgent to develop appropriate theoretical model and characteristic parameter to accurately describe the interaction between the protein and denaturant and to clearly show the features of the unfolding system.

In this work, a theoretical model is presented to illustrate the distribution, transition and thermodynamic stability of protein conformations during the denaturant-induced unfolding. This model shows the relationship between the residual activity ratio of the proteins and the denaturant concentration. Using this model, two characteristic unfolding parameters, *k*_i_ and Δ*m*_i_, which separately indicate the thermodynamic equilibrium constant for the unfolding of the proteins and the change in the number of the denaturants associated with each protein molecule between stable conformation states, can be simultaneously derived. Furthermore, using these two characteristic unfolding parameters, the distribution and transition of each stable conformation of the protein can be well described and the unfolding tendency and thermodynamic stability of each conformation can be clearly predicted over the unfolding processes.

### Theory

#### Interactions between proteins and denaturants

**Figure 1:**
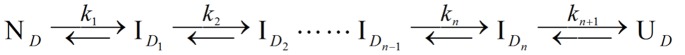
Denaturant-induced unfolding of a protein from its native state to completely unfolded state through *n* intermediate states. N*_D_*, I*_Di_* and U*_D_* denote the native state, intermediate state and completely unfolded state of a protein molecule, respectively. *k*_i_ indicates the thermodynamic equilibrium constant for the unfolding of the protein from one stable conformation state to the next. The symbol “
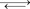
” implies that the unfolding of the protein by the denaturant is only at a local thermodynamic equilibrium under a local denaturant concentration range.

**Figure 2:**
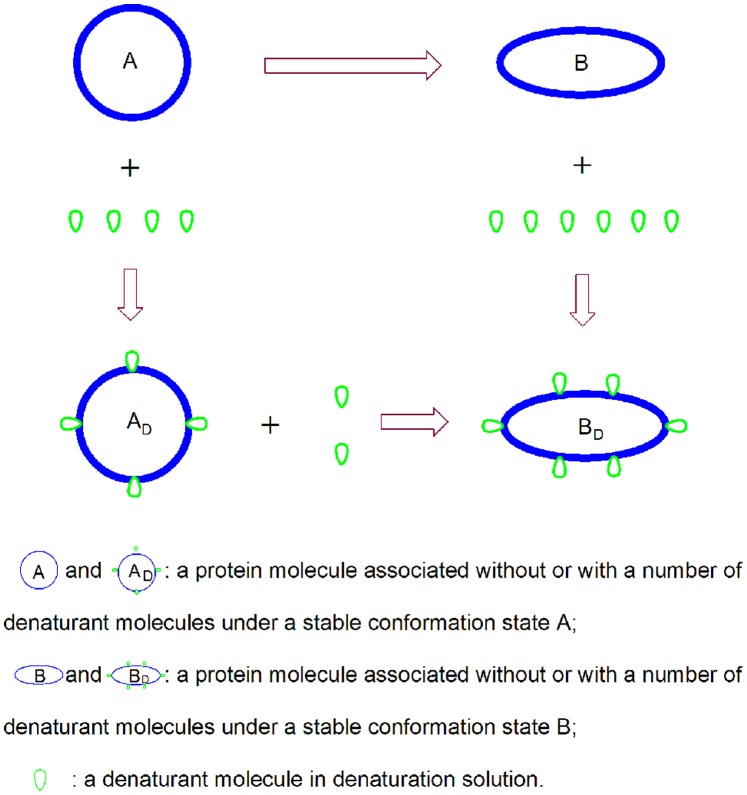
Interactions between the protein and denaturant molecules in the denaturant-induced unfolding of proteins.

For the sake of convenience, we assumed that (i) as shown in Figure 1, denaturant-induced protein unfolding usually is a progressive procedure. The protein is generally unfolded from its native state (N*_D_*) to its completely unfolded state (U*_D_*) through one or more intermediate states (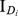) in a denaturation solution. Although the unfolding of the protein as a whole may occur through non-equilibrium thermodynamics over a wide denaturant concentration range, the protein may be in local thermodynamic equilibrium between adjacent conformational transitions under a local denaturant concentration area. Therefore, the association-dissociation interaction between the protein and denaturant can still be designated as thermodynamic equilibrium. (ii) An interaction always exists between the protein and denaturants during unfolding. The given protein may be in the N*_D_* state, I*_D_* state or U*_D_* state in the denaturation solution, and when the protein exists in different stable conformation states it can associate with different numbers of denaturant molecules (Figure 2). Therefore, when they are unfolded from one stable conformation state to another adjacent state, a change must also occur in the number of the denaturant molecules associated with the protein.

Therefore, the progressive unfolding of the protein from N*_D_* state to U*_D_* state through *n* 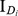 state can be expressed as:
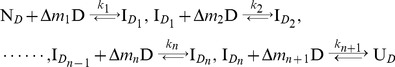
(1)where D denotes the denaturant; *k*_i_ (i = 1, 2, …, n+1) represents the thermodynamic equilibrium constants for the unfolding of the protein between stable conformation states; and Δ*m*_i_ (i = 1, 2, …, n+1) denotes the change in the number of denaturant molecules that are associated with each protein molecule between stable conformation states.

In Eqn. (1), *k*_i_ (i = 1, 2, …, n+1) can be expressed as:
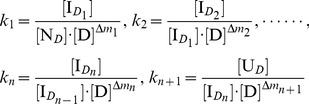
(2)where [N*_D_*], [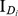] (i = 1, 2, …, n) and [U*_D_*] indicate the equilibrium concentrations (mol/L) of the native state, intermediate state and completely unfolded state of the protein in the denaturation solution, respectively, and [D] denotes the denaturant concentration (mol/L).

From Eqn. (2), we can obtain in sequence:
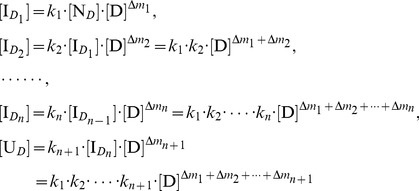
(3)


If we define the residual activity ratio (*r*) of the protein at a given denaturant concentration as the ratio of [N*_D_*] to their initial total concentration (C_t_) in the denaturation solution, then we have:
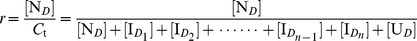
(4)


In Eqn. (4), *C*_t_ is the sum of [N*_D_*], [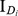] and [U*_D_*] in the denaturation solution. Therefore, substituting [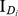] (i = 1, 2, …, n) and [U*_D_*] from Eqn. (3) in Eqn. (4) and then re-arranging it, we have:
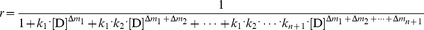
(5)


Eqn. (5) shows the dependency of the residual activity ratio of the protein on the denaturant concentration.

#### Derivation of characteristic unfolding parameters ki and Δmi

For the sake of mathematical processing convenience, we will use the three-state protein unfolding mechanism as an example to show the derivation of the characteristic unfolding parameters *k*_i_ and Δ*m*_i_. In this case, Eqn. (5) can be simplified to:
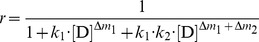
(6)where *k*_1_ and *k*_2_ indicate the thermodynamic equilibrium constants for the unfolding of the protein from N*_D_* to I*_D_* and from I*_D_* to U*_D_*, respectively. Δ*m*_1_ and Δ*m*_2_ denote the changes in the number of the denaturant molecules associated with a protein molecule during the unfolding from N*_D_* to I*_D_* and from I*_D_* to U*_D_*, respectively.

In the denaturant-induced three-state unfolding of proteins, it is not possible that each of stable conformation state is evenly distributed at different denaturant concentrations. However, it is possible that the protein mainly exists in one or two stable conformation states in a certain denaturant concentration. Therefore, Eqn. (6) can be further simplified.

- When the denaturant concentration is relatively low, the protein mainly exists in N*_D_* state and I*_D_* state. Thus, U*_D_* state can be omitted. In this case, Eqn. (6) can be simplified to:
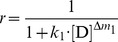
(7)when the residual activity ratio is not equal to zero or 1 and the denaturant concentration is not equal to zero. Re-arranging Eqn. (7) and taking the natural logarithm on both sides of the equation, we have:

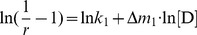
(8)As shown in Eqn. (8), in this situation, a linear relationship exists between ln(1/*r*−1) and ln[D]. Therefore, by determining the residual activity ratio over a range of relatively low denaturant concentrations, we can derive the two characteristic unfolding parameters *k*_1_ and Δ*m*_1_ from the intercept and slope of the linear regression plots of ln(1/*r*−1) versus ln[D].

- When the denaturant concentration is relatively high, the protein mainly exists in I*_D_* state and U*_D_* state. Thus, N*_D_* state can be ignored. In this situation, Eqn. (6) can be simplified to:
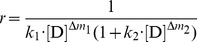
(9)when both the residual activity ratio and the denaturant concentration are not equal to zero. Re-arranging Eqn. (9) and taking the natural logarithm on both sides of the equation, we have:

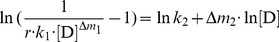
(10)As shown in Eqn. (10), a linear relationship also exists between ln[1/(*r*·*k*_1_·[D]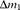)−1] and ln[D] in this case. Therefore, by determining the residual activity ratio over a range of relatively high denaturant concentrations, we can further derive the two characteristic unfolding parameters *k*_2_ and Δ*m*_2_ from the intercept and slope of the linear regression plots of ln[1/(*r*·*k*_1_·[D]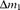)−1] versus ln[D].

In conclusion, by determining the residual activity ratio of the protein over a range of denaturant concentrations, we can separately derive the characteristic unfolding parameters *k*_1_, *k*_2_, Δ*m*_1_ and Δ*m*_2_ in the three-state unfolding of proteins induced by denaturants.

#### Distribution and transition of protein conformations

In this section, we will still use the three-state unfolding model as an example. In the denaturant-induced three-state unfolding of proteins, at a given denaturant concentration, the molar fractions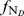, 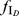
and 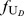
of the native state, intermediate state and completely unfolded state can be respectively expressed as:
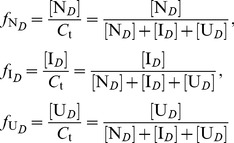
(11)


Obviously, the following relationship exists among the molar fractions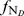, 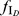
and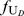:
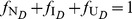
(12)


By substituting [I*_D_*] and [U*_D_*] from Eqn. (3) in Eqn. (11) and then re-arranging Eqn. (11), we have:
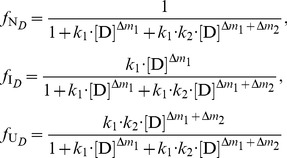
(13)


Eqn. (13) shows the effect of the denaturant concentration on the molar fractions. When the denaturant concentration approaches zero, 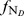
is close to 1, 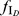
and
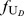
are close to zero:
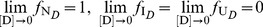
(14)


When the denaturant concentration approaches infinity, 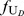 is close to 1, 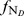
and 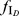 are close to zero:
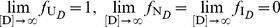
(15)


According to Eqn. (13), as long as the characteristic unfolding parameters *k*_1_, *k*_2_, Δ*m*_1_ and Δ*m*_2_ are given, we can derive the molar fractions 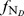, 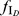
and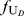 at different denaturant concentrations, and furthermore, we can describe the distribution and transition of these different conformation states.

Similarly, for the denaturant-induced two-state unfolding of proteins, the following linear relationship should exist between ln(1/*r*−1) and ln[D]:
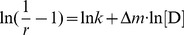
(16)


Through the linear regression plot of ln(1/*r*−1) versus ln[D], the two characteristic unfolding parameters *k* and Δ*m* can be derived from the intercept and slope, respectively. In addition, at different denaturant concentrations, the molar fractions 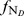
and 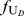 can be derived from the following equation:
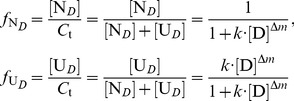
(17)


For the denaturant-induced four-state unfolding of proteins, the characteristic unfolding parameters *k*_1_, *k*_2_, *k*_3_, Δ*m*_1_, Δ*m*_2_ and Δ*m*_3_ can also be derived using the following linear regression equations:
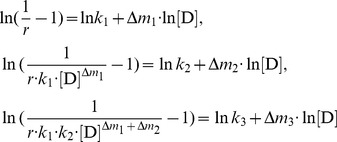
(18)


At different denaturant concentrations, the molar fractions 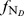,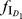,
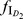
and 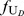 can be derived from the following equations:
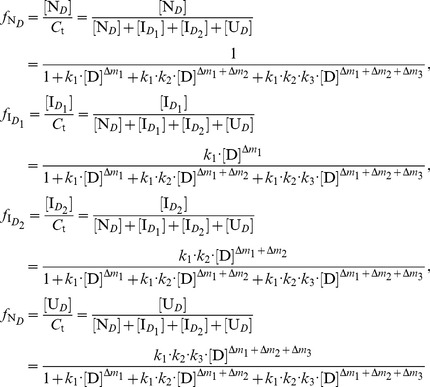
(19)


## Results

### Two-state Unfolding of Bovine Heart Cytochrome c

As shown in our previous work, the unfolding of the bovine heart cytochrome *c* induced by both guanidine hydrochloride and urea is a typical two-state process [34]. Besides, in our previous work, the structure changes of bovine heart cytochrome c during the two unfolding processes were also studied. From the results, it can be found that in the absence of denaturants, about 27% of the Trp residues in bovine heart cytochrome c are exposed to the quencher molecules; when the urea concentrations are about 1.25 and 3.0 mol/L, about 54% and 97% of the Trp residues are separately exposed to the quencher; while when the guanidine hydrochloride concentrations are about 3.0 and 6.0 mol/L, about 51% and 99% of the Trp residues are separately exposed. All the evidence indicated that the tertiary structure of bovine heart cytochrome c is destroyed with the increase of denaturants concentration, the molecule structure become loose and all the tryptophan residues originally embedded in the interior of their molecules are exposed to the surface of their molecules. Meanwhile, the size-exclusion chromatographers and native polyacrylamide gel electrophoreses results showed that during the denaturants-induced unfolding, bovine heart cytochrome c molecules exist only in a unimolecular form and their poly-molecular aggregates are not formed all along.

**Figure 3:**
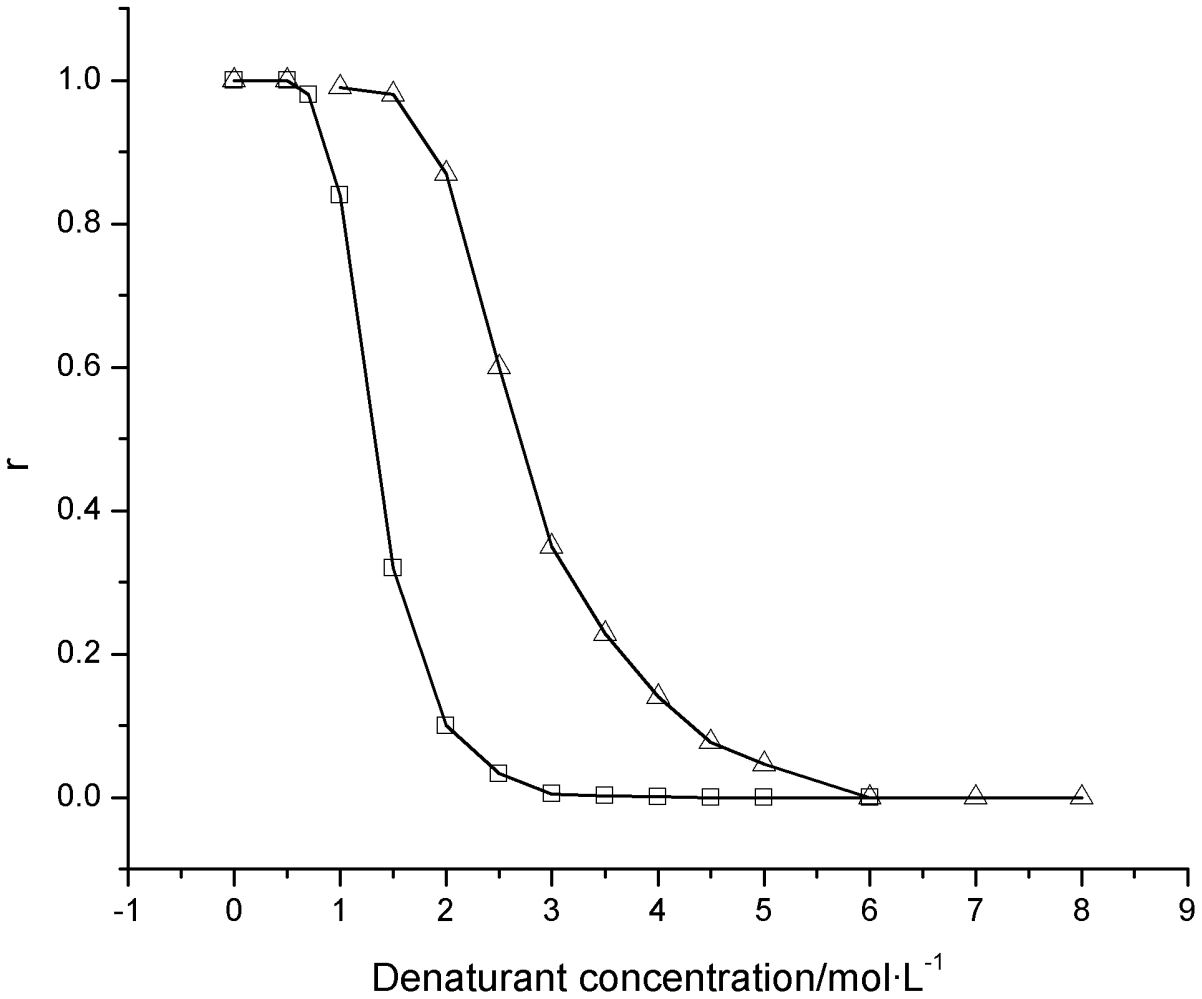
Residual activity ratios (*r*) of bovine heart cytochrome *c* exposed to different concentrations of guanidine hydrochloride or urea. □: guanidine hydrochloride; Δ: urea. The concentration of bovine heart cytochrome *c* was 0.50 mg/mL, and the experimental temperature was 25°C.

In the solution containing different concentrations of denaturants, the residual activity ratio of bovine heart cytochrome *c* was determined (Figure 3). For the two unfolding processes of bovine heart cytochrome *c*, the residual activity ratio continuously decrease from 100% to nearly 0% as the urea concentration gradually increase from 0.0 to approximately 6.0 mol/L or the guanidine hydrochloride concentration gradually increase from 0.0 to approximately 3.0 mol/L. Except when the urea concentration is equal to either 0.0 or 6.0 mol/L, the residual activity ratio of protein induced by urea is higher than that induced by guanidine hydrochloride. Therefore, it can be inferred that bovine heart cytochrome *c* molecules are more easily unfolded with guanidine hydrochloride than with urea.

**Figure 4:**
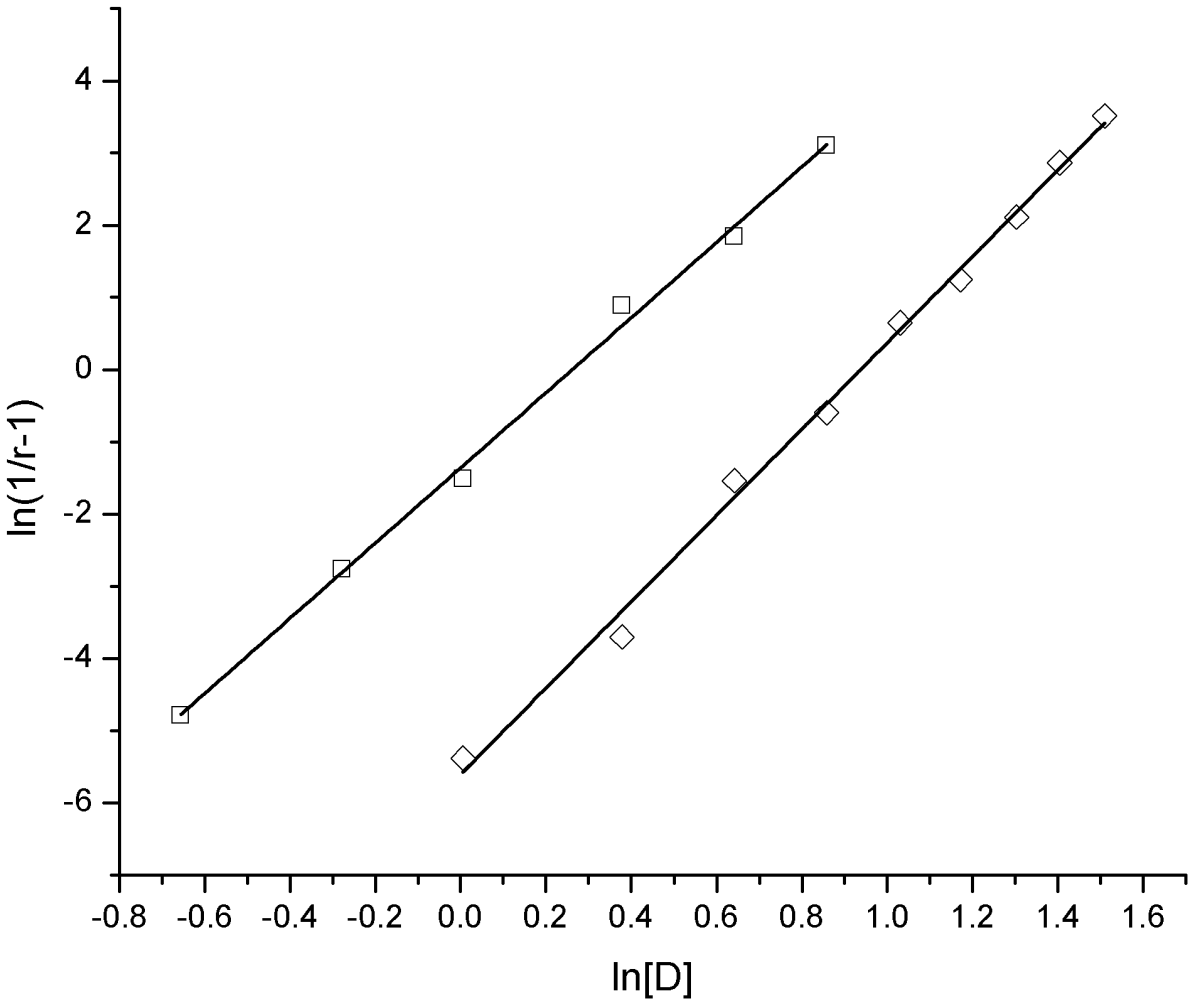
Plots of ln(1/*r*−1) vs. ln[D] for the unfolding of bovine heart cytochrome *c* induced by guanidine hydrochloride and urea. ◊: guanidine hydrochloride; □: urea.

For the two unfolding processes of bovine heart cytochrome *c*, linear regression plots of ln(1/*r*−1) vs. ln[D] were created (Figure 4) according to Eqn. (16) and the linear regression correlation coefficients (*R* ^2^) and the characteristic unfolding parameters *k* and Δ*m* were derived (Table 1). From these calculations, we determined that the good linear relationship exists between ln(1/*r*−1) and ln[D] for both the two unfolding processes and that the regression correlation coefficients (*R* ^2^) are not lower than 0.998.

**Table 1:** Regression correlation coefficients (*R* ^2^) and characteristic unfolding parameters *k* and Δ*m* for the unfolding of bovine heart cytochrome *c* induced by guanidine hydrochloride and urea.

Denaturants	conformational transitions	*R^2^*	*k/*L·mol^−1^	Δ*m*	denaturant concentration range
Guanidine hydrochloride	N*_D_* 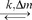 U*_D_*	0.998	2.41×10^−1^	4.85	0–6.0 L·mol^−1^
Urea	N*_D_* 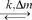 U*_D_*	0.998	3.79×10^−3^	5.53	0–8.0 L·mol^−1^

**Figure 5:**
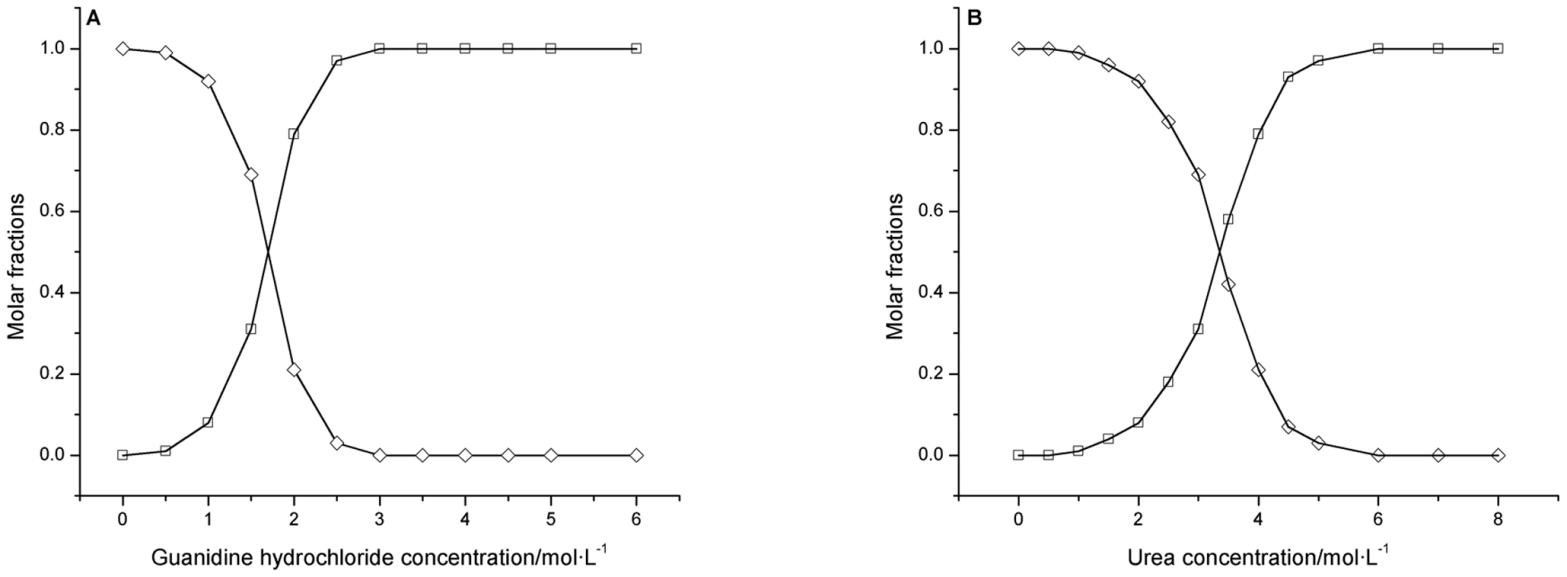
Molar fractions 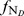 and 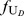 of native and completely unfolded bovine heart cytochrome *c* exposed to different concentrations of guanidine hydrochloride (A) or urea (B). ◊: native bovine heart cytochrome *c*; □: completely unfolded bovine heart cytochrome *c*.

As shown in Figures 5A and 5B, based on the above characteristic unfolding parameters *k* and Δ*m* and by using Eqn. (17), the denaturant-induced distribution and transition of native and completely unfolded bovine heart cytochrome *c* can be visualized by plotting their molar fractions 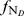
and 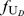
as the function of the guanidine hydrochloride or urea concentration [D]. These figures clearly show the denaturant-induced unfolding landscape of bovine heart cytochrome *c*, the percentages of N*_D_* state and U*_D_* states in the presence of different concentrations of denaturants, and how N*_D_* state gradually transforms into U*_D_* state during the unfolding processes. For example, from Figures 5A and 5B, we know that (i) both guanidine hydrochloride- and urea-induced unfolding of bovine heart cytochrome *c* are two-state processes. (ii) In the urea-induced unfolding of bovine heart cytochrome *c*, when no urea molecules exist in the solution, all of the protein exists in its native state. When the urea concentration is 1.0 mol/L, approximately 99% of the protein still exists in N*_D_* state, and only approximately 1% exists in U*_D_* state. When the urea concentration reaches approximately 5.7 mol/L, only 1% of the protein remains in N*_D_* state, and 99% exists in U*_D_* state. When the urea concentration reaches approximately 6.0 mol/L, all of the bovine heart cytochrome *c* exists in U*_D_* state. (iii) In the guanidine hydrochloride-induced unfolding of bovine heart cytochrome *c*, when the guanidine hydrochloride concentration is approximately 0.20 mol/L, only 1% of the protein exists in N*_D_* state, and 99% exists in U*_D_* state. When the guanidine hydrochloride concentration is approximately 1.5 mol/L, approximately 50% of the protein is in N*_D_* state, and 50% is in U*_D_* state. When the guanidine hydrochloride concentration reaches approximately 3.0 mol/L, all of the protein exists in U*_D_* state.

### Three-state Unfolding of Hen Egg White Lysozyme

The unfolding of hen egg white lysozyme induced by both guanidine hydrochloride and urea has been shown to be a three-state process [36]. In previous work, the results of intrinsic fluorescence anisotropy of lysozyme induced by denaturants indicated that, by raising the denaturants concentration, the flexibility and freedom degree of the six Trp residues in lysozyme molecules increase, which meant that the micro-environment where Trp residues locate in transform to a state with more loose, more flexible and smaller space occupy structure. This work also demonstrated that the deactivation precede the structure change of lysozyme molecule.

**Figure 6:**
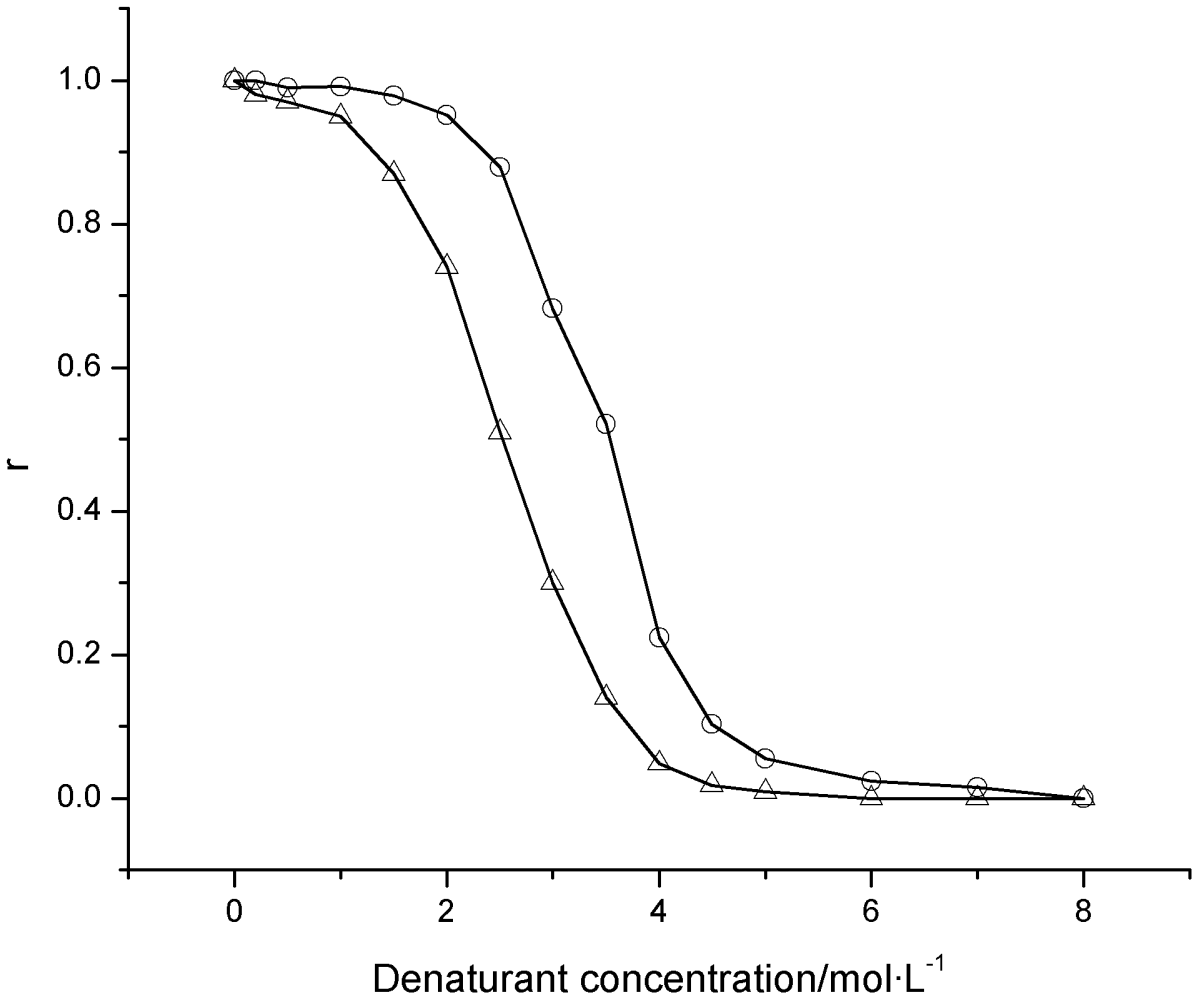
Residual activity ratios (*r*) of hen egg white lysozyme exposed to different concentrations of guanidine hydrochloride or urea. Δ: guanidine hydrochloride; ○: urea. The concentration of hen egg white lysozyme was 0.50 mg/mL, and the experimental temperature was 25°C.

In the solution containing different concentrations of denaturants, the residual activity ratio of hen egg white lysozyme was determined (Figure 6). For the two unfolding processes of hen egg white lysozyme, the residual activity ratio continuously decrease from 100% to almost 0% as the urea concentration gradually increase from 0.0 to approximately 8.0 mol/L or the guanidine hydrochloride concentration gradually increase from 0.0 to approximately 6.0 mol/L. Therefore, hen egg white lysozyme is also more easily unfolded with guanidine hydrochloride than with urea.

**Figure 7:**
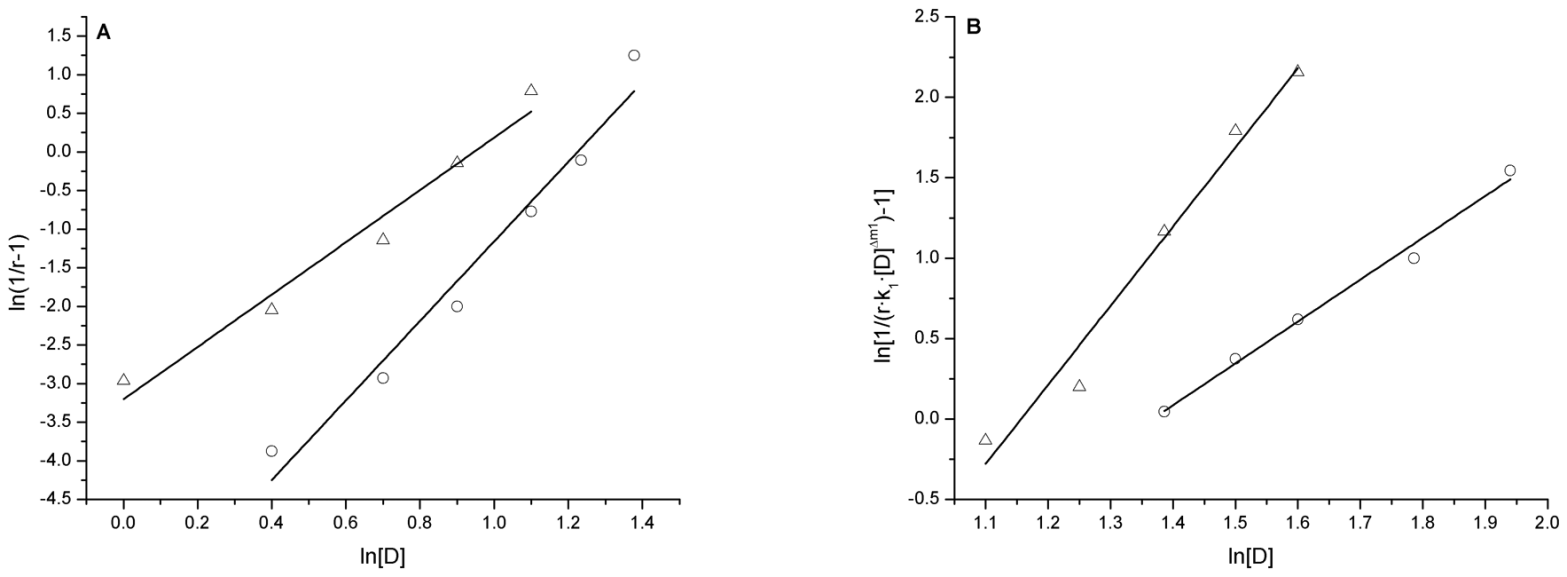
Plots of ln(1/*r*−1) vs. ln[D] (A) and ln[1/(*r*·*k*_1_·[D]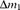)−1] vs. ln[D] (B) for the unfolding of hen egg white lysozyme induced by guanidine hydrochloride and urea. Δ: guanidine hydrochloride; ○: urea.

For the two unfolding processes of the hen egg white lysozyme, the linear regression plots of ln(1/*r*−1) vs. ln[D] and ln[1/(*r*·*k*_1_·[D]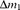)−1] vs. ln[D] were created (Figures 7A and 7B) according to Eqns. (8) and (10) and their linear regression correlation coefficients (*R* ^2^) and the characteristic unfolding parameters *k*_1_, *k*_2_, Δ*m*_1_ and Δ*m*_2_ were derived (Table 2). From these calculations, we determined that a good linear relationship exists between ln(1/*r*−1) and ln[D] and between ln[1/(*r*·*k*_1_·[D]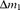)−1] and ln[D] and that all of their regression correlation coefficients are not lower than 0.985 for this process.

**Table 2:** Regression correlation coefficients (*R* ^2^) and characteristic unfolding parameters *k*_1_, *k*_2_, Δ*m*_1_ and Δ*m*_2_ for the unfolding of hen egg white lysozymes induced by guanidine hydrochloride and urea.

Denaturants	conformational transitions	*R^2^*	*k*_i_/L·mol^−1^	Δ*m*_i_	denaturant concentration range
Guanidine hydrochloride	N*_D_* 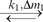 I*_D_*	0.986	4.16×10^−2^	3.41	0–2.5 L·mol^−1^
	I*_D_* 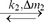 U*_D_*	0.989	3.64×10^−3^	3.86	3.5–6.0 L·mol^−1^
Urea	N*_D_* 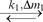 I*_D_*	0.985	1.78×10^−3^	5.14	0–2.8 L·mol^−1^
	I*_D_* 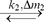 U*_D_*	0.990	2.95×10^−2^	2.58	4.5–8.0 L·mol^−1^

**Figure 8:**
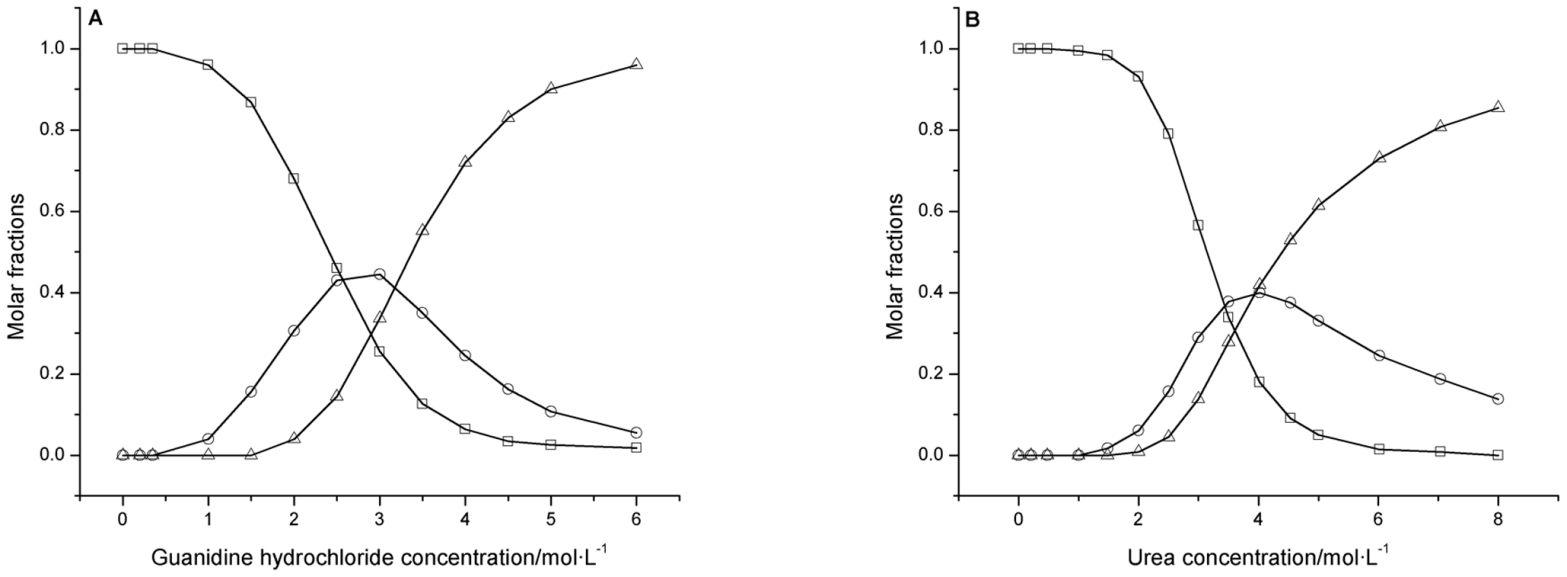
Molar fractions 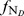, 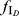 and 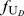 of native, intermediate and completely unfolded hen egg white lysozyme exposed to different concentrations of guanidine hydrochloride (A) or urea (B). □: native egg white lysozyme; ○: intermediate egg white lysozyme; Δ: completely unfolded egg white lysozyme.

The molar fractions 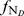, 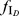 and 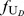 of hen egg white lysozyme at different concentrations of denaturants were derived (Figures 8A and 8B) according to Eqn. (13). These results indicate that (i) both the unfolding of guanidine hydrochloride- and urea-induced hen egg white lysozyme are three-state processes, and the protein molecule can only be transformed from its N*_D_* state to U*_D_* state through an intermediate state. (ii) In the guanidine hydrochloride solution, the hen egg white lysozyme molecule is first unfolded from its N*_D_* state to I*_D_* state as the guanidine hydrochloride concentration increases to approximately 3.0 mol/L and then is further unfolded from I*_D_* state to U*_D_* state as the guanidine hydrochloride concentration further increases to approximately 6.0 mol/L. The maximum molar fraction of the protein in I*_D_* state is approximately 0.42. (iii) In the urea solution, the egg white lysozyme molecule is first unfolded from its N*_D_* state to I*_D_* state when the urea concentration increases to approximately 4.0 mol/L and then is further unfolded from I*_D_* state to U*_D_* state when the urea concentration further increases to approximately 8.0 mol/L. The maximum molar fraction of hen egg white lysozyme in I*_D_* state is approximately 0.40.

### Four-state Unfolding of Bovine Carbonic Anhydrase b

The guanidine hydrochloride-induced unfolding of bovine carbonic anhydrase *b* has been shown to be a four-state process [38]. To the structure change of carbonic anhydrase molecule during the guanidine hydrochloride-induced unfolding, it was found that the first intermediate has no rigid tertiary structure but is almost as compact as the native protein, and it meets all the usual requirements of the molten globule state. The second intermediate, which is less compact than the molten globule state but much more compact than the unfolded state, represents a novel, “pre-molten globule” state. The CD results indicated that the secondary structure of carbonic anhydrase is destroyed in two phases: first, upon transition between molten globule and pre-molten globule states and then upon a complete unfolding of protein molecules, besides, both the two intermediates have substantial secondary structure. The ANS binding results suggested that the pre-molten globule state binds ANS about five times more weakly than the molten globule state. And the size-exclusion chromatography results illustrated that the pre-molten globule state is reasonably compact.

**Figure 9:**
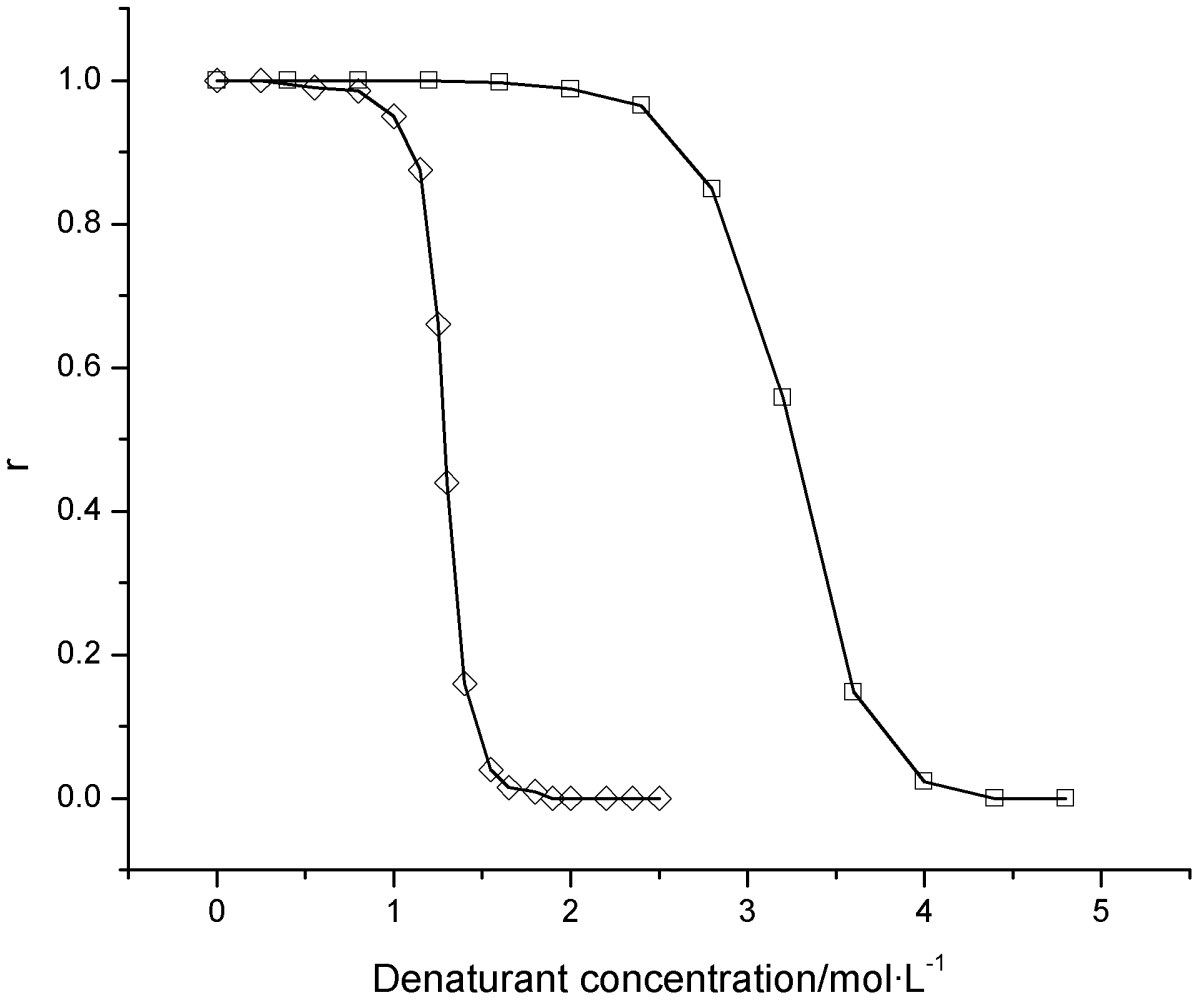
Residual activity ratios (*r*) of bovine carbonic anhydrase *b* exposed to different concentrations of guanidine hydrochloride or urea. ◊: guanidine hydrochloride; □: urea. The concentration of bovine carbonic anhydrase *b* was 0.50 mg/mL, and the experimental temperature was 25°C.

The residual activity ratio of bovine carbonic anhydrase *b* exposed to different concentrations of denaturants was determined now (Figure 9). For the two unfolding processes of bovine carbonic anhydrase *b*, the residual activity ratio of protein continuously decrease from 100% to nearly 0% as the urea concentration gradually increase to approximately 4.4 mol/L or the guanidine hydrochloride concentration gradually increase to approximately 2.2 mol/L. Therefore, bovine carbonic anhydrase *b* is also more easily unfolded with guanidine hydrochloride than with urea.

Similarly, for the guanidine hydrochloride-induced four-state unfolding of bovine carbonic anhydrase *b*, plots of the residual activity ratio of protein vs. the denaturant concentration were also created according to Eqn. (18), and their linear regression correlation coefficients (*R* ^2^) and the characteristic unfolding parameters *k*_1_, *k*_2_, *k*_3_, Δ*m*_1_, Δ*m*_2_ and Δ*m*_3_ were also derived (Table 3). As shown in Table 3, all of the regression correlation coefficients are not lower than 0.985.

**Table 3:** Regression correlation coefficients (*R* ^2^) and characteristic unfolding parameters *k*_1_, *k*_2_, *k*_3_, Δ*m*_1_, Δ*m*_2_ and Δ*m*_3_ for the unfolding of bovine carbonic anhydrase *b* induced by guanidine hydrochloride.

Conformational transitions	*R^2^*	*k*_i_ */*L·mol^−1^	Δ*m*_i_	guanidine hydrochloride concentration range
N*_D_* 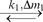 MG*_D_*	0.988	1.35×10^−2^	10.02	0–1.3 L·mol^−1^
MG*_D_* 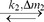 PMG*_D_*	0.997	6.91×10^−4^	14.70	1.4–1.8 L·mol^−1^
PMG*_D_* 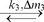 U*_D_*	0.992	7.71×10^−5^	17.30	1.9–2.4 L·mol^−1^

**Figure 10:**
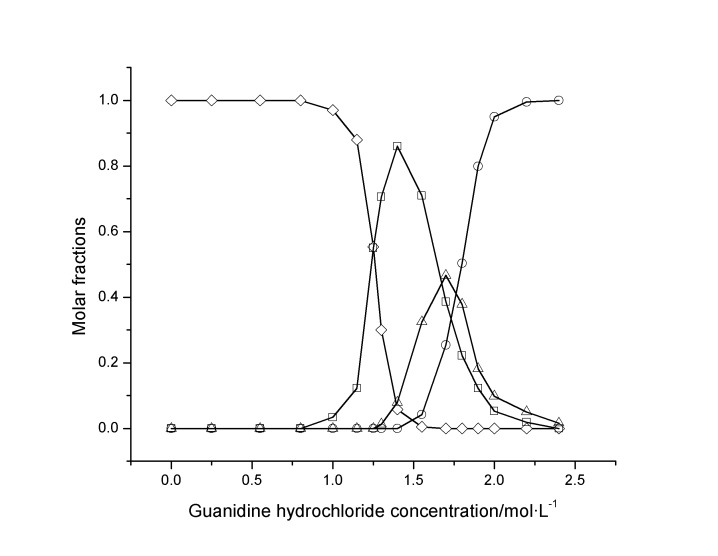
Molar fractions 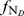, 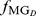, 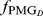 and 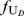 of native, molten globule, pre-molten globule and completely unfolded bovine carbonic anhydrase *b* exposed to different concentrations of guanidine hydrochloride. ◊: native bovine carbonic anhydrase *b*; □: molten globule bovine carbonic anhydrase *b*; Δ: pre-molten globule bovine carbonic anhydrase *b*; ○: completely unfolded bovine carbonic anhydrase *b*.

Based on Eqn. (19), we can also derive the molar fractions 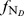, 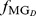, 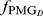 and 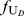 of bovine carbonic anhydrase *b* molecules at different concentrations of guanidine hydrochloride (Figure 10). Through these calculations, we found that (i) the unfolding of guanidine hydrochloride-induced bovine carbonic anhydrase *b* is a typical four-state process and that bovine carbonic anhydrase *b* can only be transformed from its N*_D_* state to U*_D_* state through two sequential intermediate states, the I*_MG_* state and the I*_PMG_* state. (ii) In the guanidine hydrochloride solution, bovine carbonic anhydrase *b* is first unfolded from its N*_D_* state to MG*_D_* state as the guanidine hydrochloride concentration increases to approximately 1.4 mol/L. The protein is then further unfolded from MG*_D_* state to its PMG*_D_* state as the guanidine hydrochloride concentration further increases to approximately 1.7 mol/L. Finally, bovine carbonic anhydrase *b* transforms from its PMG*_D_* state to U*_D_* state as the guanidine hydrochloride concentration further increases to approximately 2.2 mol/L. The concentrations at which the number of molecules in the molten MG*_D_* state and the PMG*_D_* state reaches its maximum are approximately 1.40 and 1.7 mol/L guanidine hydrochloride, respectively. These results are approximately consistent with those from a previously published study [38].

### Unfolding of other Proteins

In all of the unfolding processes described above, we were able to derive the characteristic unfolding parameters *k*_i_ and Δ*m*_i_ by exploiting the relationship between the residual activity ratio (*r*) and the denaturant concentration ([D]). Furthermore, through these characteristic unfolding parameters, we were able to describe the distribution and transition profiles of each stable conformational state at different denaturant concentrations. However, we can also derive the characteristic unfolding parameters *k*_i_ and Δ*m*_i_ through the distribution and transition data of the denaturant-induced unfolding of the proteins.

For example, in the denaturant-induced three-state unfolding of hen egg white lysozyme, when the denaturant concentration is relatively low, the protein mainly exists in its N*_D_* state and I*_D_* state, and U*_D_* state can be omitted. In this case, using Eqn. (13), we obtain:
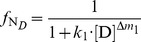
(20)


When the molar fraction 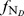 of egg white lysozyme in N*_D_* state is not equal to zero or 1 and by re-arranging Eqn. (20) and taking the natural logarithm, we have:
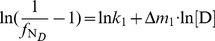
(21)


When the denaturant concentration is relatively high, hen egg white lysozyme mainly exists in its I*_D_* state and U*_D_* state, and its N*_D_* state can be ignored. In this case, through Eqn. (13), we obtain:
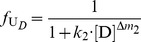
(22)


Similarly, when the molar fraction 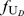 of egg white lysozyme in U*_D_* state is not equal to zero or 1 and by re-arranging Eqn. (22) and taking the natural logarithm, we also obtain:
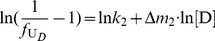
(23)


Eqns. (21) and (23) show that in the denaturant-induced three-state unfolding of hen egg white lysozyme, a linear relationship exists between 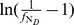 and ln[D] and between 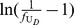 and ln[D]. From their intercepts and slopes, the characteristic unfolding parameters *k*_1_, Δ*m*_1_ and *k*_2_, Δ*m*_2_ can be simultaneously derived.

Using the distribution and transition data for the unfolding of other protein molecules induced by denaturants in the literature, as shown in Table 4, we derived their regression correlation coefficients (*R* ^2^) and the characteristic unfolding parameters *k*_i_ and Δ*m*_i_. These data included the three-state unfolding of *β*-lactoglobulin induced by 0–80% (v/v) methanol [41], the four-state unfolding of the apo form of myristoylated NCS-1 induced by 0–6.0 mol/L guanidine hydrochloride and the apo form of non-myristoylated NCS-1 induced by 0–5.0 mol/L guanidine hydrochloride [42], the four-state unfolding of adenylated tslig induced by 0–5.0 mol/L guanidine hydrochloride and de-adenylated tslig induced by 0–7.0 mol/L guanidine hydrochloride [43], the four-state unfolding of *β*-lactamase induced by 0–2.0 mol/L guanidine hydrochloride [44] and the quasi-five-state unfolding of rabbit muscle creatine kinase induced by 0–6.0 mol/L guanidine hydrochloride [45]. As shown in Table 4, all of their regression correlation coefficients are greater than 0.985.

**Table 4:** Regression correlation coefficients (*R* ^2^) and characteristic unfolding parameters *k*_i_ and Δ*m*_i_ for the unfolding of proteins induced with different denaturants.

Conformational transitions	*R^2^*	*k*_i_ */*L·mol^−1^	Δ*m*_i_	denaturant concentration range
The three-state unfolding of *β*-lactoglobulin induced by methanol				
N*_D_* 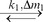 MG*_D_*	0.990	8.61×10^−9^	9.17	0–25% (V/V)
MG*_D_* 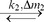 U*_D_*	0.988	1.51×10^−5^	5.39	50–70% (V/V)
The four-state unfolding of the apo form of myristoylated NCS-1 induced by guanidine hydrochloride				
N*_D_* 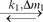 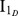	0.991	5.55	2.52	0–1.5 L·mol
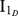 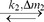 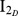	0.997	3.50×10^−5^	9.39	1.5–4.0 L·mol
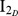 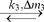 U*_D_*	0.995	1.78×10^−8^	9.65	4.5–7.0 L·mol
The four-state unfolding of the apo form of non-myristoylated NCS-1 induced by guanidine hydrochloride				
N*_D_* 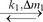 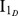	0.993	1.44×10^1^	4.50	0–1.0 L·mol
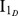 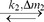 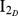	0.998	3.78×10^−5^	9.41	1.0–3.5 L·mol
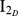 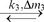 U*_D_*	0.997	4.57×10^−6^	10.40	4.8–7.0 L·mol
The four-state unfolding of adenylated tslig induced by guanidine hydrochloride				
N*_D_* 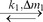 MG*_D_*	0.996	4.92×10^−2^	4.72	0–2.5 L·mol
MG*_D_* 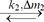 PMG*_D_*	0.991	4.29×10^−6^	9.29	2.5–3.4 L·mol
PMG*_D_* 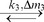 U*_D_*	0.987	9.92×10^−9^	13.62	3.6–4.6 L·mol
The four-state unfolding of de-adenylated tslig induced by guanidine hydrochloride				
N*_D_* 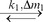 MG*_D_*	0.988	1.13×10^−1^	3.53	0–2.5 L·mol
MG*_D_* 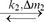 PMG*_D_*	0.991	3.06×10^−11^	16.79	2.5–4.0 L·mol
PMG*_D_* 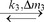 U*_D_*	0.994	6.68×10^−10^	13.74	5.0–7.0 L·mol
The four-state unfolding of *β*-lactamase induced by guanidine hydrochloride				
N*_D_* 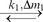 MG*_D_*	0.987	1.99×10^1^	2.66	0–0.3 L·mol
MG*_D_* 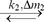 PMG*_D_*	0.987	5.29×10^1^	2.17	0.5–0.8 L·mol
PMG*_D_* 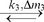 U*_D_*	0.989	4.25	4.70	1.0–2.0 L·mol
The quasi-five-state unfolding of rabbit muscle creatine kinase induced by guanidine hydrochloride				
N*_D_* 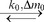 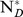	0.986	2.77×10^5^	4.70	0–0.2 L·mol^−1^
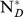 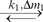 MG*_D_*	0.991	7.95×10^3^	8.99	0.3–0.6 L·mol^−1^
MG*_D_* 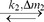 PMG*_D_*	0.988	5.30×10^−1^	9.69	0.6–2.2 L·mol^−1^
PMG*_D_* 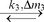 U*_D_*	0.995	2.53×10^−4^	12.91	2.2–5.8 L·mol^−1^

## Discussion

### Fitting of the Theoretical Model

In all of the above fitting analyses, including that of Eqn. (16) to the two-state unfolding data of bovine heart cytochrome *c*, Eqns. (8) and (10) to the three-state unfolding data of hen egg white lysozyme, Eqn. (18) to the four-state unfolding data of bovine carbonic anhydrase *b* and Eqns. (21) and (23) to the three-state unfolding data of hen egg white lysozyme, at least 10 sets of experimental data were used to fit these equations and all of the regression correlation coefficients were greater than 0.985. Therefore, it can be concluded that this theoretical model can be used to fit the residual activity ratio and the distribution and transition data of proteins exposed to different denaturant concentrations with a 99% degree of confidence.

### Characteristic Unfolding Parameters k_i_ and Δm_i_

As shown in the mathematical expression of the theoretical model, Eqn. (5) includes two characteristic unfolding parameters *k*_i_ and Δ*m*_i_. Both *k*_i_ and Δ*m*_i_ only depend on the inherent characteristics of the protein and denaturant molecules but not relate to the denaturant concentration. *k*_i_ is the thermodynamic equilibrium constant for the denaturant-induced unfolding of proteins, and it shows the unfolding tendency of protein from one stable conformation state to the next. Δ*m*_i_ is the difference in the number of denaturant molecules associated with a protein molecule between stable conformation states, and it shows the ability of a denaturant to unfold a protein in a given unfolding system. Therefore, it can be inferred that a relatively larger *k*_i_ or a relatively smaller Δ*m*_i_ means that it is easier for the protein transition from one stable conformation state to the next; whereas a relatively smaller *k*_i_ or a relatively larger Δ*m*_i_ means that it is harder for this transition to occur.

For example, as shown in Table 1 for the two-state unfolding of bovine heart cytochrome *c*, the characteristic unfolding parameter *k* is 3.79×10^−3^ and 2.41×10^−1^ L·mol^−1^ for urea and guanidine hydrochloride, respectively. The value for urea is approximately 64 times that for guanidine hydrochloride. Meanwhile,the Δ*m* is 5.53 and 4.85 for urea and guanidine hydrochloride respectively. Both *k* and Δ*m* show that the guanidine hydrochloride-induced conformational transition of bovine heart cytochrome *c* from its native state to its completely unfolded state is easier than the urea-induced transition.

In another example, as shown in Table 2 for the three-state unfolding of hen egg white lysozyme induced by guanidine hydrochloride, the characteristic unfolding parameters *k*_1_ and *k*_2_ are 4.16×10^−2^ and 3.64×10^−3^ L·mol^−1^, respectively. The value of *k*_1_ is approximately 11 times that of *k*_2_. Meanwhile, Δ*m*_1_ and Δ*m*_2_ are 3.41 and 3.86 respectively. These results show that the conformational transition of hen egg white lysozyme induced by guanidine hydrochloride from its native conformation state (N*_D_*) to its intermediate conformation state (I*_D_*) is easier than from its intermediate conformation state (I*_D_*) to its completely unfolded conformation state (U*_D_*). For the three-state unfolding of hen egg white lysozyme induced by urea, the characteristic unfolding parameters *k*_1_ and *k*_2_ are 1.78×10^−3^ and 2.95×10^−2^ L·mol^−1^ while Δ*m*_1_ and Δ*m*_2_ are 5.14 and 2.78, respectively. These results show that the urea-induced conformational transition of hen egg white lysozyme from N*_D_* to I*_D_* is harder than the transition from I*_D_* to U*_D_*. However, for the three-state unfolding of the hen egg white lysozymes, the total characteristic unfolding parameter *k*_t_, which is equal to the product of the characteristic unfolding parameters *k*_1_ and *k*_2_, is 1.51×10^−4^ and 5.25×10^−5^ L·mol^−1^ for guanidine hydrochloride and urea, respectively. While the total characteristic unfolding parameter Δ*m*_t_, which is equal to the sum of the characteristic unfolding parameters Δ*m*_1_ and Δ*m*_2_, is 7.27 and 7.72 for the two unfolding processes, respectively. Both of these parameters show that the conformational transition of hen egg white lysozyme from its native stable conformation state to its completely unfolded state induced by guanidine hydrochloride is easier than that induced by urea.

And as shown in Tables 3 and 4, similar situations also exist for the unfolding of other proteins induced by denaturants.

**Figure 11:**
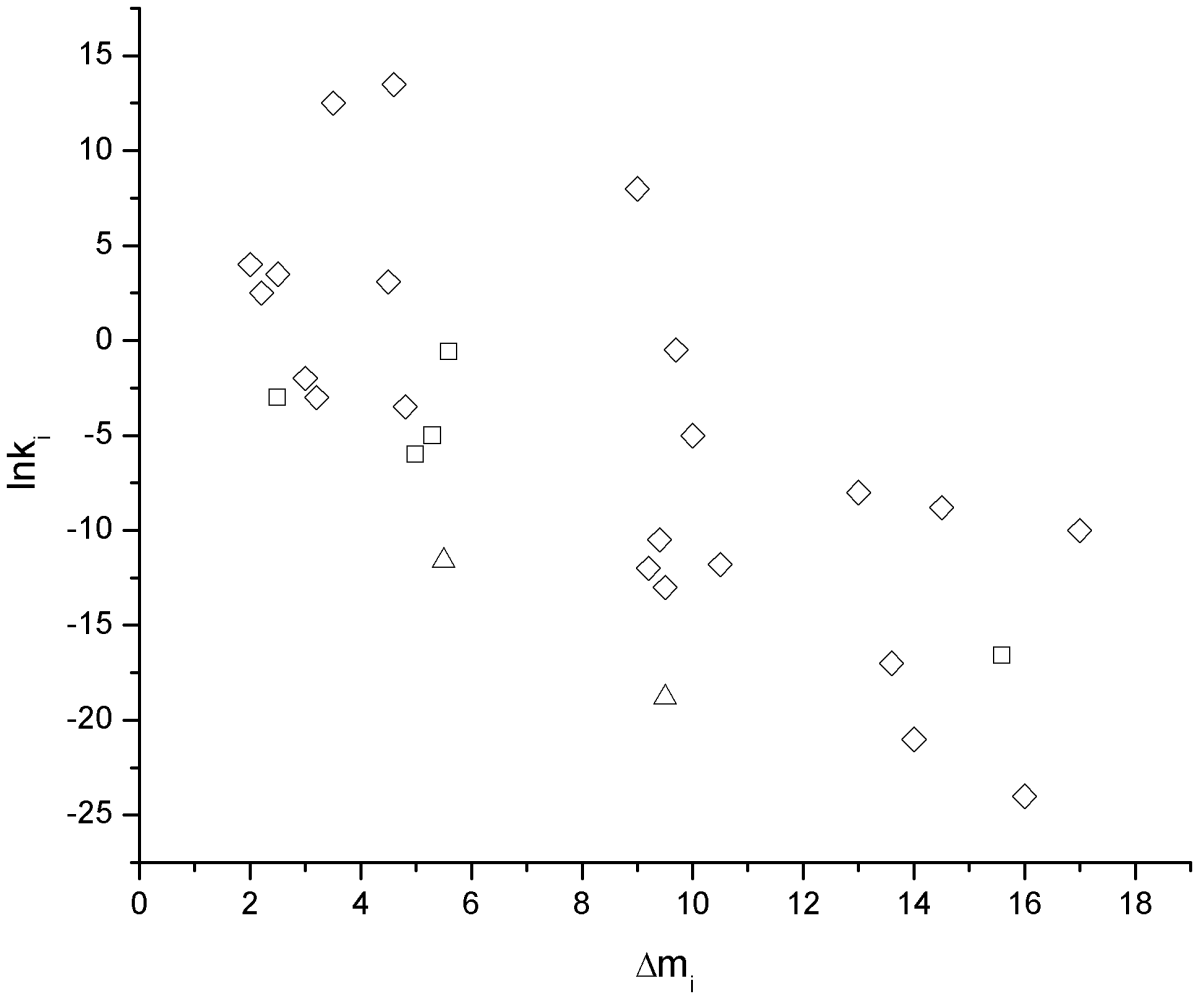
Plots of ln*k*_i_ vs. Δ*m*_i_ for the unfolding of some proteins induced by denaturants. ◊: guanidine hydrochloride; □: urea; Δ: methanol.

In addition, through carefully analyzing and comparing *k*_i_ and Δ*m*_i_ in Tables 1 to 4, we found that a reverse change exists between the characteristic unfolding parameters in all of these unfolding systems, in other words, in each step of the conformation state transition, a relatively large *k*_i_ is always followed by a relatively small Δ*m*_i_, and vice versa. From the above results, we can further predict whether a linear regression relationship definitely exists between the characteristic unfolding parameters *k*_i_ and Δ*m*_i_. However, as shown in Figure 11 and Eqn. (24), when linear regression plots of ln*k*_i_ vs. Δ*m*_i_ were created, no statistically linear relationship existed between the characteristic unfolding parameters:
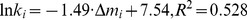
(24)


This result means that *k*_i_ and Δ*m*_i_ are two independent variables. They separately show the characteristic features of the denaturant-induced unfolding of proteins from two different aspects.

From both the fitting analyses of the mathematical expressions to the experimental data and the physical meanings of the characteristic unfolding parameters, it may be concluded that this theoretical model can be used to effectively describe the unfolding of proteins induced by denaturants.

### Unfolding Tendency and Thermodynamic Stability

The characteristic unfolding parameters *k*_i_ and Δ*m*_i_ in this model are the thermodynamic equilibrium constant for the denaturant-induced unfolding of proteins and the change in the number of the denaturant molecules that are associated with a protein molecule between two successive stable conformation states, respectively. Therefore, for a given unfolding system, we can estimate the unfolding tendency and thermodynamic stability of each of its conformation states.

In Tables 2, 3 and 4, through carefully comparing the characteristic unfolding parameters, it can be found that, in most of the examined unfolding processes, the *k_i_* in the subsequent transition step is smaller than that in the previous step, and the Δ*m*_i_ is larger in the next transition step than in the previous transition step. However, these trends are not the case for the urea-induced three-state unfolding of hen egg white lysozyme; the methanol-induced three-state unfolding of *β*-LG; the guanidine hydrochloride-induced four-state unfolding of de-adenylated Tslig; and the guanidine hydrochloride-induced four-state unfolding of *β*-lactamase. These data indicate that in most denaturant-induced protein unfolding processes, the unfolding of the protein becomes increasingly harder with each transition step.

At a given temperature (T), the free energy change (Δ*G*_U_) in the denaturant-induced unfolding of a protein from its stable conformation state (A) to its adjacent stable conformation state (B) can be expressed as:
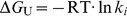
(25)where R indicates the gas constant, and *k*_i_ denotes the thermodynamic equilibrium constant for the unfolding from its state A to its adjacent state B. Using Eqn. (25), we can estimate the stability of the protein in each stable conformation states during the unfolding process. If *k*_i_ is more than 1, then the free energy change Δ*G*_U_ is negative, indicating that the unfolding of the protein from state A to its adjacent state B is spontaneous and that the protein is more stable in state B than in state A. If *k*_i_ is less than 1, then the free energy change Δ*G*_U_ is positive, indicating that the unfolding of the protein from state A to state B is not spontaneous and that the protein is more stable in state A than in state B. If *k*_i_ is close to 1, then the free energy change Δ*G*_U_ is close to zero, indicating that the unfolding of the protein from state A to state B is approximately equal to its refolding from state B to state A and that the state A is approximately as stable as state B.

As shown in Tables 1 to 4, in most denaturant-induced unfolding processes, *k_i_* is less than 1, and consequently, Δ*G*_U_ is positive. This is the case except for the guanidine hydrochloride-induced four-state transitions of the apo-NCS-1 and four-state transitions of the apo-non-myristoylated NCS-1(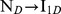); the guanidine hydrochloride-induced four-state transition of *β*-lactamase(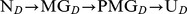); and the guanidine hydrochloride-induced quasi-five-state transition of rabbit muscle creatine kinase(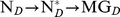, 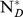:dimeric state with a rigid tertiary structure). Therefore, in most denaturant-induced protein unfolding processes, the protein is more unstable in its next adjacent stable conformation state than in its previous stable conformation state.

In summary, a theoretical model was presented to show the dependence of the residual activity ratio of proteins on the molar denaturant concentration in the denaturant-induced unfolding, the distribution, transition, unfolding tendency and thermodynamic stability of protein conformations can be quantitatively described by the two characteristic unfolding parameters *k*_i_ and Δ*m*_i_ in this model. In most denaturant-induced unfolding processes of proteins, the unfolding becomes increasingly harder in next transition step and the protein becomes more unstable as it attains each successive stable conformation. This work presents a useful method for people to study the unfolding of proteins and may be used to describe the unfolding and refolding of other biopolymers induced by denaturants, inducers, *etc*.

## Materials and Methods

### Reagents and Chemicals

Bovine heart cytochrome *c*, bovine carbonic anhydrase *b* and dried *Micrococcus lysodeikticus* cells were purchased from Sigma. Hen egg white lysozyme (20000 units/mg), yeast cytochrome *c*, reduced and oxidized glutathione (highly pure), urea (highly pure) and guanidine hydrochloride (highly pure) were purchased from Shanghai Sangon Biological and Technological Service Co., Ltd. All other reagents were of analytical grade without further purification, and all solutions were prepared in bi-distilled water and passed through a 0.22 µm filter.

### Unfolding of Bovine Heart Cytochrome *c*

In previous work, we determined the intrinsic fluorescence emission spectrum, fluorescence phase diagram, and fluorescence quenching and deactivation profile for the unfolding of bovine heart cytochrome *c* induced by urea and guanidine hydrochloride and found that the unfolding of bovine heart cytochrome *c* induced by both guanidine hydrochloride and urea was a typical two-state process [34].

According to Darley-Usmar *et al.* [35], the activity of bovine heart cytochrome *c* at different concentrations of guanidine hydrochloride or urea was determined using the Clark oxygen-electrode method, in which the oxidase activity was measured by determining the oxygen concentration in solution. Yeast cytochrome *c* was used as reference to measure the difference in voltage (ΔmV). The characteristic unfolding parameters *k* and Δ*m* for bovine heart cytochrome *c* under different concentrations of guanidine hydrochloride and urea were derived using Eqn. (16), and the molar fractions 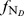 and 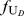 of the native state (N*_D_*) and completely unfolded state (U*_D_*) were obtained with Eqn. (17).

### Unfolding of Hen Egg White Lysozyme

Using the “phase diagram” method of fluorescence, Yang *et al*. found that the unfolding of hen egg white lysozyme induced by both guanidine hydrochloride and urea was a typical three-state process [36].

In the study of Rozema and Gellman [37], the activity of hen egg white lysozyme exposed to different concentrations of guanidine hydrochloride and urea was assayed at 25°C by calculating the decrease in the absorbance of the reaction solution at 450 nm. The reaction was carried out by adding 0.1 mL of the enzyme solution to 1.0 mL of a 0.25 mg/mL *Micrococcus lysodeikticus* suspension in 0.06 mol/L potassium phosphate (pH 6.2). One activity unit corresponded to an absorbance decrease of 0.0026 per minute. Using different concentrations of guanidine hydrochloride and urea, the characteristic unfolding parameters *k*_1_ and Δ*m*_1_, and *k*_2_ and Δ*m*_2_ for hen egg white lysozyme were derived using Eqns. (8) and (10). In addition, the molar fractions 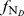, 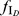 and 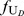 of the native state (N*_D_*), intermediate state (I*_D_*) and completely unfolded state (U*_D_*) were obtained using Eqn. (13).

### Unfolding of Bovine Carbonic Anhydrase b

Using far UV circular dichroism spectroscopy, fluorescence spectroscopy and size-exclusion chromatography, Uversky and Ptitsyn found that the unfolding of bovine carbonic anhydrase *b* induced by guanidine hydrochloride is a four-state process [38].

In the study of Brownell *et al*. [39], the esterase activity of bovine carbonic anhydrase *b* was measured using the rate of *p*-nitrophenyl acetate cleavage, which was monitored by the increase in absorption at 348 nm. The reaction was initiated by adding 0.2 mL of the 0.1 mg/mL protein stock solution to 20.0 mL of the reaction mixture containing 0.18 mg/mL *p*-nitrophenyl acetate. Using different concentrations of guanidine hydrochloride, the characteristic unfolding parameters *k*_1_ and Δ*m*_1_, *k*_2_ and Δ*m*_2_, and *k*_3_ and Δ*m*_3_ for bovine carbonic anhydrase b were derived using Eqn. (18). Additionally, the molar fractions 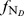, 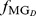, 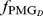 and 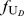 of the native state (N*_D_*), molten globule state (MG*_D_*), pre-molten globule state (PMG*_D_*) and completely unfolded state (U*_D_*) were obtained using Eqn. (19).

### Protein Concentration

Protein concentration was determined using the Bradford method [40]. The absorbance at 595 nm was measured after the addition of Coomassie Brilliant Blue G-250 to the protein sample, and bovine serum albumin was used to determine the standard curve.
